# Exploring the link between synesthesia and lucid dreaming through perceptual presence

**DOI:** 10.3389/fpsyg.2026.1733841

**Published:** 2026-02-26

**Authors:** Eiko Matsuda, Eiko Matsuda

**Affiliations:** 1 Graduate School of Science and Technology, Keio University, Yokohama, Japan; 2 Department of Social Psychology, Faculty of Sociology, Toyo University, Tokyo, Japan

**Keywords:** consciousness, counterfactuals, dreaming, lucid dream, perceptual presence, sensorimotor contingencies, synesthesia

## Abstract

This study investigates links between synesthesia and lucid dreaming via perceptual presence and counterfactual-richness (abundant possible sensorimotor contingencies). We hypothesized that synesthetes would report more frequent lucid dreams because enhanced counterfactual-richness facilitates dream control and clarity. We surveyed 616 adults using a synesthesia self-report, the Lucidity and Consciousness in Dreams scale (LuCiD), and the Big-5 inventory (TIPI-J). Cluster analysis validated four synesthesia subtypes– Language-Color, Ordinal Linguistic Personification (OLP), Spatial Sequence, and Visualized sensation–consistent with prior work. Regression analyses revealed type-specific effects on lucid dreaming: perceptual synesthesia (Visualized sensation, Spatial Sequence) robustly promoted lucid-dream facets–especially control, and also insight, dissociation, and positive emotion–whereas conceptual synesthesia (Language-Color, OLP) showed negative interactions with Openness and Extraversion, thereby attenuating lucid-dream experiences. Personality analyses further confirmed positive associations between lucid dreaming and Openness and Extraversion, aligning with previous literature. We interpret perceptual synesthesia as an expression of excessive counterfactual-richness that enhances perceptual presence and sensorimotor contingencies during dreaming. These findings both clarify qualitative differences within synesthetic experience and suggest a new direction for understanding synesthesia and lucid dreaming as interconnected cognitive phenomena.

## 1 Introduction

Synesthesia is a phenomenon wherein a sensory stimulus induces a different type of sensory perception. For example, viewing letters can evoke color sensations, or hearing sounds can induce perceptions of shapes, demonstrating diverse combinations of sensory modalities between the inducing stimulus (inducer) and the resulting perception (concurrent) (Ward, 2013). The primary characteristics of synesthesia include (1) stable correspondences maintained over long periods (temporal consistency), (2) idiosyncratic stimulus-perception combinations unique to individuals (e.g., one synesthete may perceive violin sounds as red, while another may perceive them as green; idiosyncrasy), (3) automatic and rapid occurrence of induced perceptions (automaticity), and (4) occurrence limited to a subset of the population (Ward, 2013; Deroy and Spence, 2013; Chiou et al., 2013). In particular, grapheme-color synesthesia is the most extensively studied, appearing in approximately 1% of the population (Ward, 2013). Additionally, grapheme-color synesthesia has been reported to exhibit relatively stable prevalence from childhood to adulthood (Simner et al., 2009; Simner and Bain, 2013; Meier et al., 2014), while qualitative changes such as reduced temporal consistency and diminished color vividness occur with age (Meier et al., 2014; Simner et al., 2017; Pfeifer et al., 2017).

One of the challenges in synesthesia research is its unclear relation to other general cognitive phenomena. As synesthesia is relatively rare, it is often understood as a special perceptual phenomenon, making it difficult to integrate within general cognitive models. Given that synesthesia involves interactions between multiple sensory modalities, it has been compared to crossmodal correspondences and sound symbolism (Deroy and Spence, 2013). Crossmodal correspondences refer to broadly shared, natural associations between senses, such as “higher pitches associated with brighter colors” (Mondloch and Maurer, 2004). Sound symbolism, exemplified by the “bouba/kiki effect,” refers to associations between linguistic sounds and meanings (Köhler, 1929; Ramachandran and Hubbard, 2001). These phenomena share crossmodal elements with synesthesia but differ notably by lacking the idiosyncrasy characteristic of synesthesia (Deroy and Spence, 2013).

In this study, we explore potential links between synesthesia and lucid dreaming. Lucid dreams refer to experiences in which individuals become aware that they are dreaming during the dream itself and may, in some cases, intentionally manipulate dream content (Stickgold et al., 2000; Laberge, 1985; Voss et al., 2009; Barrett, 2017). Synesthesia is clearly distinct from dreaming; nevertheless, both synesthesia and lucid dreams share the vivid perception of sensations or situations that are not actually present.

Here, we introduce the concept of *perceptual presence*, proposing that synesthesia involves a higher-than-normal perceptual presence. Perceptual presence refers to the sense that something exists in reality and is thought to be determined by counterfactual-richness (Noë, 2004, 2006; Seth, 2014). Counterfactual richness refers to how richly an experience contains *potentialities* of sensorimotor contingencies (SMC) – that is, expectations of how sensation would change if one were to act in a certain way (Noë, 2004, 2006; Seth, 2014). For example, compare a tomato in front of you with a tomato depicted in a picture. Even if the two produced very similar retinal images, with the real tomato you can anticipate rich SMC potentials; such as “if I rotate my face, I will see its side or back,” “if I move closer, the shading will change,” and “if I pick it up, I will obtain tactile and olfactory information” (*counterfactually-rich*). By contrast, for the painted tomato, no matter how you change your viewpoint, SMCs such as “directly seeing the back of the painted tomato” do not, in principle, hold; thus, the richness of potential SMCs you can anticipate is relatively narrow (*counterfactually-poor*). The *potentialities* of SMCs refer to the fact that the action actually being performed is not required (counterfactual); rather, it concerns whether such potential SMCs are felt as available within experience (Noë, 2004, 2006; Seth, 2014). For instance, you may feel a potential SMC, in which you can grab and rotate the actual tomato, without actually doing the action. Seth (2014) argues that when these counterfactually-rich predictions are in place, perceptual presence is enhanced; perceptual content is experienced as “part of the real world” and as “continuous with the world” (Noë, 2004, 2006; Seth, 2014).

On this view, we can treat the concurrent sensations that arise in synesthesia as additional sensations layered on top of ordinary perception. If so, for the same stimulus, the richness of potential SMCs—“if I were to move like this, my sensations should change like that”—can increase, and the experience can involve higher *counterfactually richness*. Following Seth's (2014) framework, synesthesia may therefore strengthen perceptual presence insofar as it involves higher counterfactual richness .

In lucid dreams–particularly those with high controllability–the possibility of SMCs increases significantly, as dreamers can intentionally alter or control the dream content, enhancing counterfactual-richness. For instance, attempts like “moving/transforming objects,” “trying to fly” or “trying to switch scenes,” successively create new SMCs within the dream. Accordingly, in lucid dreams, particularly those with high controllability, the possibility of SMCs is expanded, rendering the experience more counterfactually rich. As a result, the degree to which the dream experience is felt as “really there”—that is, its perceptual presence—may be enhanced (Seth, 2014).

If synesthesia indeed results in excessive counterfactual-richness, synesthetes might similarly experience heightened counterfactual-richness within their dreams. Consequently, it can be hypothesized that synesthetes would experience controllable lucid dreams more frequently. In fact, previous research suggests that synesthetes have a higher incidence of lucid dreaming. According to Khallieva et al. (2022), 80.6% of grapheme-color synesthetes reported experiencing lucid dreams, compared to 53.1% of a control group. However, this study has several limitations. First, it exclusively examined grapheme-color synesthesia despite the existence of multiple synesthesia types, leaving unexplored the relationship between other synesthetic forms and lucid dreaming. Additionally, it did not clearly outline the psychological and cognitive mechanisms underlying the facilitation of lucid dreaming by synesthesia.

Both synesthesia and lucid dreaming are known to be associated with personality traits. Synesthesia, in particular, shows a relatively strong correlation with the Big-5 personality trait of Openness, and some studies report a weak association with Neuroticism, although results for the latter have been inconsistent (Rouw and Scholte, 2016; Shi and Matsuda, 2024). Lucid dreaming has also been notably associated with Openness (Hess et al., 2017), suggesting that personality traits might moderate the relationship between synesthesia and lucid dreaming.

Based on these considerations, we examined the association between synesthesia subtypes and the frequency of lucid dreaming–particularly controllable lucid dreaming–while accounting for personality traits. Specifically, after entering Big-Five personality traits as covariates, we tested whether endorsement of the four major synesthesia types–Language-color (colors accompanying letters/numbers and related linguistic stimuli; Baron-Cohen, 1996), Ordinal Linguistic Personification (OLP; personification attributes such as personality and/or gender assigned to numbers, weekdays, and other ordinal sequences; Simner and Holenstein, 2007), Spatial sequence (experiencing sequences such as numbers, times, height, temperature as having a continuous spatial layout; Eagleman, 2009), and Visualized sensation (a broad “sensation-to-vision” type in which sensory inputs such as sounds, taste, pain and emotions elicit visual concurrents such as colors and shapes; Chiou et al., 2013; Ward et al., 2006)—was associated with a higher frequency of lucid dreams. In addition, we exploratorily evaluated whether personality traits (e.g., Openness) might amplify or attenuate these associations by testing interactions between synesthesia subtype indicators and personality traits.

We conducted a survey of 808 young adults using a synesthesia self-report questionnaire to assess synesthesia types, the Japanese version of the TIPI (TIPI-J; Oshio et al., 2012) to measure personality traits, and the LuCiD (the Lucidity and Consciousness in Dreams scale) to evaluate the frequency and quality of lucid dreams (Ward and Simner, 2022; Voss et al., 2013).

While the standard method for assessing synesthesia is the temporal consistency test (Eagleman et al., 2007), this study employed a self-report method due to the necessity of comprehensively evaluating multiple synesthesia types and examining their relationship with lucid dreaming in a large sample. Although self-report methods may yield a higher proportion of synesthetes compared to consistency tests, their validity has been supported by previous research (Rouw and Scholte, 2016; Ward and Simner, 2022).

The primary objective of this study is to understand synesthesia not as a unique perceptual phenomenon but in relation to more general cognitive phenomena, including lucid dreaming. To position synesthesia within a general cognitive framework, it is crucial to capture the diverse and dynamic experiences characteristic of synesthesia. Therefore, this study adopted the self-report method, which effectively captures a broader range of self-reported synesthesia-like experiences that are challenging to detect with temporal consistency tests, and examined these associations within a large sample. Through this approach, the study aims to demonstrate that synesthesia can be understood within the context of broader cognitive models, including lucid dreaming. Ultimately, this research proposes a new perspective, positioning synesthesia along a continuum of cognitive phenomena rather than viewing it as an isolated phenomenon.

## 2 Materials and methods

### 2.1 Participants

Participants in this study were 808 undergraduate students enrolled in a university psychology course on “Dreams and Sleep” within the general psychology curriculum. Of these, 616 students (241 males, 371 females; mean age = 19.44 years, SD = 1.295) provided complete responses to all questionnaires and were included in the analyses. This study was approved by the Ethics Committee of Toyo University (Approval number: P240021). All participants provided written informed consent in accordance with the Declaration of Helsinki.

### 2.2 Questionnaire

The following three questionnaires were utilized in this study:

- Synesthesia self-report questionnaire: this questionnaire was adapted from the synesthesia self-report survey developed by Ward and Simner (2022) to comprehensively and uniformly capture a wide range of synesthetic inducer–concurrent pairings. Participants reported whether they experienced induced sensations (concurrents), such as color, shape, spatial arrangement, personality, gender, taste, or touch, in response to diverse inducing stimuli such as letters, numbers, sounds, pain, time, and sequences (see Supplementary Table S1).

Typically, synesthesia is identified using behavioral diagnostic approaches that assess the consistency of inducer–concurrent mappings across time (e.g., consistency-based tests; Eagleman et al., 2007). However, this study used a self-report method to broadly assess both visual and non-visual synesthetic experiences, including spatial arrangement, personality, taste, and touch. Although self-report methods may yield higher estimates of synesthesia prevalence than methods based on temporal consistency (Rouw and Scholte, 2016; Ward and Simner, 2022) and may include individuals whose experiences are not temporally consistent, previous studies have demonstrated the validity of self-reports. For example, self-reported synesthetic profiles have been consistently correlated with objective behavioral indicators such as sensory hypersensitivity and imagery abilities (Rouw and Scholte, 2016). Furthermore, consistent cognitive profiles of synesthetes based on self-report data have also been established (Ward and Simner, 2022). Thus, while acknowledging its limitations, including the potential for inflated prevalence rates, the use of self-report methods is justified given the aims of this research.

Previous self-report studies have shown that synesthetic experiences cluster into multiple types, among which four types—Language – color, OLP, Spatial sequence, and Visualized sensation—occur with particularly high frequency. Accordingly, following prior work, the present study focused on these four major types (Ward and Simner, 2022). Other less prevalent types were also assessed in the questionnaire (see Supplementary Table S1), but were excluded from the main inferential analyses due to low endorsement rates and limited statistical power.

- Japanese Version of the TIPI (TIPI-J): the TIPI-J is a concise and efficient scale for assessing personality traits based on the Big Five dimensions: Extraversion, Neuroticism (emotional instability), Openness, Conscientiousness, and Agreeableness. Each dimension is measured by two items (one positively worded and one negatively worded) using a 7-point Likert scale (total of 10 items) (Oshio et al., 2012). Although Openness and Neuroticism were of a priori interest based on prior work, all Big Five traits were included in the exploratory analyses.
- LuCiD (the Lucidity and Consciousness in Dreams scale): Dream lucidity was assessed using the LuCiD (Voss et al., 2013). Participants were asked to briefly describe any lucid dream experiences during the past year and then completed 28 items covering eight subscales – insight (recognition of dreaming), control (ability to manipulate dream content), thought (logical reasoning), realism (dream realism), memory (memory access within dreams), dissociation (sense of detachment in dreams), negative emotion, and positive emotion. Items were rated on a 6-point scale (1 = strongly disagree, 6 = strongly agree) and recoded to the original 0–5 scoring prior to computing subscale and total scores (0 = strongly disagree, 5 = strongly agree; Voss et al., 2013). A Japanese version was created via back translation for use in this study. The objective was to examine in detail how specific synesthesia types and personality traits influence different aspects of lucid dreaming experiences.

Using the questionnaire survey described above, this study aims to comprehensively and multidimensionally analyze the relationship between synesthesia and lucid dreaming, thereby offering new insights into the task of situating synesthesia within a continuum of general cognitive phenomena.

### 2.3 Procedure

The survey was conducted during psychology classes focused on “Dreams and Sleep.” The questionnaires were distributed by the researchers responsible for the classes, and participants responded individually. The survey was conducted across three sessions: personality traits (TIPI-J) in the first, lucid dreaming experiences (LuCiD) in the second, and the synesthesia self-report questionnaire in the third. Each questionnaire session took approximately 10 min.

### 2.4 Statistical analysis

All analyses were conducted in R (version 4.3.2). Only participants with complete responses to all questionnaires were included in the analyses (*n* = 616; listwise deletion).

#### 2.4.1 Coding of synesthesia types

Based on the synesthesia self-report questionnaire (Supplementary Table S1), four major synesthesia types were operationalized as follows: Type 1 (Language–color; Q1), Type 2 (OLP/Personification; Q6), Type 3 (Spatial sequence; Q5), and Type 4 (Visualized sensation; Q2–Q3). Each type was coded as a binary indicator (0 = absent, 1 = present). A participant was coded as present for a given type if they answered “Yes” to the corresponding question and endorsed at least one relevant inducer–concurrent item within that type (endorsement of all items was not required). Participants could endorse multiple types.

#### 2.4.2 Reliability and correlational analyses

Internal consistency of the LuCiD total score and each subscale was evaluated using Cronbach's α. Pearson's correlation coefficients were computed between LuCiD scores (total and subscales) and Big Five personality traits (TIPI-J) to assess their associations.

#### 2.4.3 Cluster analysis

Following prior work using the same self-report approach, synesthetic experiences can be grouped into multiple clusters, of which the present study focused on the four most prevalent types (Language–color, OLP, Spatial sequence, and Visualized sensation; Ward and Simner, 2022). Therefore, hierarchical cluster analysis was conducted as a construct validity check to examine whether item-level response patterns reproduced the expected four-group structure. We used Euclidean distance and Ward's minimum-variance method, and set the number of clusters a priori to 4 (*k* = 4) based on our predefined focus on these four major types.

#### 2.4.4 Regression analyses

Multiple linear regression analyses were conducted with the LuCiD total score and each LuCiD subscale score as dependent variables. Predictors included the four synesthesia-type indicators and the Big Five trait scores. Interaction terms between synesthesia types and personality traits were also considered to test moderation. All variables were standardized (*z*-scored) prior to regression analyses. Model selection was performed using an Akaike Information Criterion (AIC)–based stepwise procedure (step function in the stats package), which balances model fit and complexity. Statistical significance was evaluated using two-tailed tests with α = 0.05. Because predictors were selected using an AIC-based stepwise procedure, we report coefficients for all predictors retained in the final models, regardless of whether they reach the conventional significance threshold.

## 3 Results

### 3.1 Synesthesia endorsement rate and cluster structure

Responses to the Synesthesia Self-Report Questionnaire were summarized for the four primary synesthesia types targeted in the present study (Type 1: Language–color, Type 2: OLP, Type 3: Spatial sequence, Type 4: Visualized sensation).

**Figure 1:**
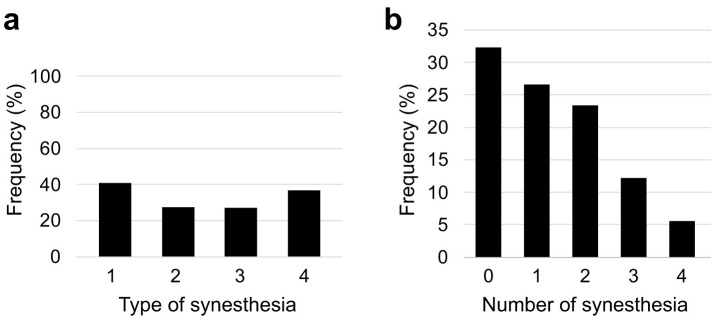
Endorsement rate and distribution of synesthesia types based on the Synesthesia Self-Report Questionnaire. **(a)** Percentage of participants reporting at least one of the four primary synesthesia types (Type 1: Language-color, Type 2: OLP, Type 3: Spatial-sequence, Type 4: Visualized sensation). **(b)** Frequency distribution (%) of the number of synesthesia types simultaneously reported by participants (0 indicates no endorsement of any of the four primary types).

Figure 1a shows the endorsement rate (self-report) of each synesthesia type and Figure 1b the distribution of the number of synesthesia types endorsed by participants. A participant was considered to endorse a given synesthesia type if they responded “yes” to at least one item belonging to that type; participants could endorse multiple types. A value of zero in panel (**b**) indicates participants who did not endorse any of the four primary synesthesia types. Accordingly, the overall endorsement rate (68.0%) reflects the endorsement of at least one synesthesia-like experience.

To examine the structure of item-level synesthesia responses, hierarchical cluster analysis (Euclidean distance and Ward's minimum-variance method, *k* = 4) was conducted. The dendrogram and cluster assignment are shown in Supplementary Figure S1. The four-cluster solution yielded the following clusters: (i) Language–color (e.g., letters–color, numbers–color), (ii) OLP (e.g., numbers–personality, days–personality), (iii) spatial sequence (e.g., numbers–spatial arrangement, months–spatial arrangement), and (iv) Visualized sensation (e.g., music–color, emotion–color). In this solution, the item “Posture–color” was assigned to the Language–color cluster (Supplementary Figure S1).

### 3.2 LuCiD internal consistency and correlations with personality traits

Internal consistency of the LuCiD was evaluated using Cronbach's α coefficients (Table 1). Cronbach's α was 0.79 for the LuCiD total score. Subscale α values were 0.87 (Insight), 0.82 (Control), 0.70 (Thought), 0.66 (Realism), 0.62 (Memory), 0.69 (Dissociation), 0.86 (Positive Emotion), and 0.87 (Negative Emotion).

**Table 1:** Internal consistency (Cronbach's α) and descriptive statistics for the LuCiD total score and subscales (*N* = 616).

**Scale**	***k***	***M***	***SD***	**Range**	**Cronbach's α**
LuCiD total	28	1.63	0.84	0.00–4.75	0.79
Insight	6	1.67	1.29	0.00–5.00	0.87
Control	5	1.31	1.19	0.00–5.00	0.82
Thought	3	1.74	1.27	0.00–5.00	0.70
Realism	3	2.09	1.27	0.00–5.00	0.66
Memory	4	1.40	1.02	0.00–4.75	0.62
Dissociation	3	1.05	1.12	0.00–5.00	0.69
Positive emotion	2	2.19	1.58	0.00–5.00	0.86
Negative emotion	2	2.21	1.59	0.00–5.00	0.87

Subscale scores and the total LuCiD score were computed as mean item scores of their constituent items. *k* denotes the number of items in each subscale. *M* and *SD* are mean item scores on the 0–5 scale. Items were rated on a 6-point scale and recoded to match the original LuCiD scoring (0 = strongly disagree, 5 = strongly agree).

Table 1 also reports descriptive statistics for the LuCiD total score and each subscale (mean item scores on the 0–5 scale). Because lucidity was operationalized as continuous LuCiD scores, participants were not dichotomized into “lucid” vs. “non-lucid” dreamers; accordingly, we do not report a single categorical count of lucid dreamers. Instead, the distribution of scores provides an overview of individual differences in lucid-dream features (e.g., Insight: *M* = 1.67, *SD* = 1.29, range = 0.00–5.00; LuCiD total: *M* = 1.63, *SD* = 0.84, range = 0.00–4.75).

Pearson correlation coefficients between LuCiD scores (total and subscales) and Big Five personality traits (TIPI-J) are shown in Table 2. The LuCiD total score was positively correlated with Extraversion (*r* = 0.15, *p* < 0.001) and Openness (*r* = 0.08, *p* < 0.05). Control and Thought were also positively correlated with Extraversion (Control: *r* = 0.13, *p* < 0.01; Thought: *r* = 0.11, *p* < 0.01) and Openness (Control: *r* = 0.09, *p* < 0.05; Thought: *r* = 0.11, *p* < 0.01).

**Table 2:** Correlation coefficients and *p*-values between LuCiD scale (total and subscales) and Big-5 personality traits (TIPI-J).

**LuCiD scale**	**Extraversion**	**Agreeableness**	**Conscientiousness**	**Neuroticism**	**Openness**
Total score	0.15^***^	−0.00	0.05	0.04	0.08^*^
Insight	0.12^**^	0.03	0.08	0.06	0.06
Control	0.13^**^	−0.04	0.07	0.03	0.09^*^
Thought	0.11^**^	0.05	0.02	0.05	0.11^**^
Realism	0.05	0.04	−0.00	0.06	0.02
Memory	0.16^***^	−0.04	0.03	0.03	0.09^*^
Dissociation	0.07	−0.09^*^	0.02	-0.06	0.01
Negative Emotion	−0.03	−0.03	−0.08^*^	0.00	−0.01
Positive Emotion	0.12^**^	0.01	0.04	−0.01	−0.01

^*^p < 0.05, ^**^p < 0.01, ^***^p < 0.001.

### 3.3 Regression analyses predicting LuCiD outcomes

Multiple linear regression analyses were conducted for the LuCiD total score and each LuCiD subscale (Tables 3, 4). Predictors retained in the final models were selected using an AIC-based stepwise procedure. Standardized coefficients are reported.

**Table 3:** Results of multivariate regression analysis for LuCiD overall score.

**Dependent variable**	**Predictor**	**Estimate**	**Std. Error**	***t*-value**	***p*-value**
LuCiD overall score	Type 1: Language-color	−0.038	0.040	−0.94	0.347
	Type 2: OLP	−0.016	0.041	−0.39	0.700
	Type 3: Spatial sequence	0.104	0.041	2.55	0.011^*^
	Type 4: Visualized sensation	0.236	0.041	5.75	<0.001^***^
	Extraversion (P1)	0.105	0.039	2.70	0.007^**^
	Openness (P5)	0.053	0.038	1.37	0.171
	Type1 × Extraversion	−0.100	0.038	−2.60	0.010^**^
	Type2 × Openness	−0.079	0.039	−2.01	0.044^*^
Adjusted *R*^2^	0.103			

Predictors were selected using an AIC-based stepwise procedure. ^*^p < 0.05, ^**^p < 0.01, ^***^p < 0.001.

**Table 4:** Results of multiple regression analyses for LuCiD subscales.

**Dependent variable**	**Predictor**	**Estimate**	**Std. Error**	***t*-value**	***p*-value**
Insight (d1)	Type 1 (Language–color)	−0.092	0.040	−2.28	0.023^*^
	Type 4 (Visualized sensation)	0.152	0.041	3.66	<0.001^***^
	Extraversion (P1)	0.082	0.040	2.06	0.040^*^
	Type 1 × Extraversion	−0.107	0.039	−2.73	0.007^**^
Adjusted *R*^2^	0.061			
Control (d2)	Type 1 (Language–color)	−0.089	0.040	−2.19	0.029^*^
	Type 3 (Spatial sequence)	0.103	0.041	2.48	0.013^*^
	Type 4 (Visualized sensation)	0.197	0.042	4.74	<0.001^***^
	Extraversion (P1)	0.097	0.039	2.47	0.014^*^
	Type 2 (OLP) × Extraversion	−0.081	0.038	−2.14	0.033^*^
Adjusted *R*^2^	0.076			
Thought (d3)	Type 4 (Visualized sensation)	0.185	0.041	4.49	<0.001^***^
	Openness (P5)	0.091	0.039	2.33	0.020^*^
	Type 1 × Extraversion	−0.107	0.040	−2.63	0.009^**^
	Type 4 × Extraversion	0.093	0.042	2.22	0.027^*^
Adjusted *R*^2^	0.073			
Realism (d4)	Type 4 (Visualized sensation)	0.105	0.042	2.50	0.013^*^
Adjusted *R*^2^	0.024			
Memory (d5)	Type 4 (Visualized sensation)	0.180	0.041	4.37	<0.001^***^
	Extraversion (P1)	0.123	0.039	3.14	0.002^**^
Adjusted *R*^2^	0.071			
Dissociation (d6)	Type 3 (Spatial sequence)	0.093	0.042	2.24	0.026^*^
	Type 4 (Visualized sensation)	0.178	0.041	4.31	<0.001^***^
	Agreeableness (P2)	−0.089	0.041	−2.19	0.029^*^
	Type 2 (OLP) × Openness (P5)	−0.119	0.040	−2.95	0.003^**^
Adjusted *R*^2^	0.067			
Negative emotion (d7)	Type 1 (Language–color)	0.097	0.041	2.33	0.020^*^
	Type 4 × Extraversion	0.089	0.041	2.18	0.030^*^
	Type 4 × Agreeableness	−0.084	0.041	−2.06	0.040^*^
Adjusted *R*^2^	0.030			
Positive emotion (d8)	Type 3 (Spatial sequence)	0.114	0.041	2.79	0.006^**^
	Type 4 (Visualized sensation)	0.146	0.042	3.50	<0.001^***^
	Extraversion (P1)	0.093	0.040	2.30	0.022^*^
Adjusted *R*^2^	0.055			

Each LuCiD subscale served as a dependent variable. Predictors were selected using an AIC-based stepwise procedure. This table reports only statistically significant standardized coefficients (*p* < 0.05) from the final models; full coefficients for all predictors retained in the final models (including non-significant terms) are provided in Supplementary Tables 2, 3.

^*^ *p* < 0.05, ^**^p < 0.01, ^***^p < 0.001.

Table 3 summarizes the final model for the LuCiD total score (adjusted *R*^2^ = 0.103). Positive coefficients were observed for Type 3 (Spatial sequence), Type 4 (Visualized sensation), and Extraversion, whereas negative coefficients were observed for the interaction terms Type 1 × Extraversion and Type 2 × Openness.

For the eight LuCiD subscales, Table 4 reports only statistically significant standardized coefficients (*p* < 0.05) from the AIC-selected final models (adjusted *R*^2^ range: 0.024–0.076). Full coefficients for all predictors retained in the AIC-selected models (including non-significant terms) are provided in Supplementary Tables S2, S3. Across subscales, Type 4 was retained with positive coefficients in each model, with significant positive coefficients for most of the subscales, including Insight, Control, Thought, Realism, Memory, Dissociation, and Positive Emotion. Type 3 was retained in the final models for all outcomes except Negative Emotion and showed consistently positive coefficients, reaching conventional significance in the models for Control, Dissociation, and Positive Emotion. Type 1 was retained in multiple subscale models, showing negative coefficients for Insight and Control and a positive coefficient for Negative Emotion.

Personality traits contributed both as main effects and as moderators. Extraversion showed positive main effects on several outcomes, including Insight, Control, Memory, and Positive Emotion. Openness showed a positive main effect specifically for Thought (logical reasoning within dreams), whereas Agreeableness showed a negative association with Dissociation.

In addition, several interaction terms indicated moderation by personality traits: Type 1 × Extraversion was retained with negative coefficients for Insight and Thought; Type 2 × Extraversion was retained with a negative coefficient for Control; and Type 2 × Openness was retained with a negative coefficient for Dissociation. Type 4 also showed interactions with personality traits in specific affective outcomes (Type 4 × Extraversion for Negative Emotion; Type 4 × Agreeableness for Negative Emotion).

**Figure 2:**
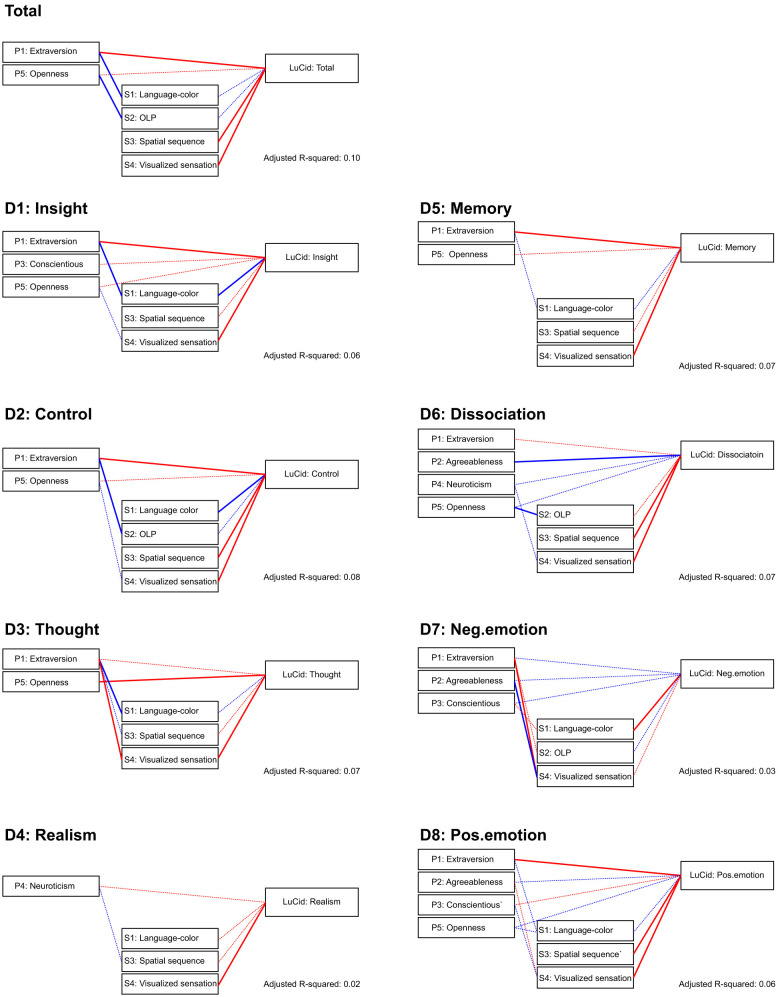
Conceptual diagram summarizing the standardized coefficients from the AIC-selected final regression models for the LuCiD total score and each LuCiD subscale (d1–d8). Red lines indicate positive coefficients and blue lines indicate negative coefficients. Solid lines indicate statistically significant coefficients (*p* < 0.05), whereas dashed lines indicate non-significant coefficients (*p*≥0.05). Adjusted *R*^2^ for each final model is shown at the bottom right.

Figure 2 provides a schematic overview of the direction of standardized coefficients for the final models. Because final models were selected using an AIC-based stepwise procedure, some predictors were retained despite not reaching *p* < 0.05; these coefficients are shown as dashed lines in Figure 2 and reported in full in Supplementary Tables S2, S3.

## 4 Discussion

This study aimed to reconsider synesthesia, traditionally treated as a special perceptual phenomenon, within the context of broader cognitive phenomena. Building on the framework of perceptual presence and counterfactual-richness (Noë, 2004, 2006; Seth, 2014), we proposed that self-reported synesthetic experiences may involve excessive counterfactual-richness and that this property could extend to dreaming, potentially relating to lucid dreaming experiences—particularly to the ability to intentionally manipulate dream content (Control). Prior work suggests that synesthetes may report lucid dreaming more frequently (Khallieva et al., 2022); however, previous evidence has primarily focused on grapheme–color synesthesia and has not clarified mechanisms across synesthesia types. Therefore, we examined type-specific associations between synesthesia and lucid dreaming and tested whether personality traits modulate these associations (Rouw and Scholte, 2016; Hess et al., 2017).

We conducted a questionnaire survey in Japanese undergraduate students (*n* = 616) using a comprehensive synesthesia self-report questionnaire (Ward and Simner, 2022), the LuCiD scale for lucid dreaming experiences (Voss et al., 2013), and the Japanese version of the TIPI for Big Five personality traits (Oshio et al., 2012). Personality traits were included as covariates and potential moderators through interaction terms, consistent with the moderation hypothesis described in the Introduction.

The results indicated clear heterogeneity across synesthesia types. In the regression models, Type 4 (Visualized sensation) and Type 3 (Spatial sequence) showed positive coefficients for the LuCiD total score and appeared as positive predictors across multiple LuCiD subscales (including Control), whereas Type 1 (Language–color) and Type 2 (OLP) showed weaker or negative effects in specific models and were most evident through interactions with personality traits (Tables 3, 4, Figure 2).

In the following sections, we interpret this type-specific pattern using the conceptual-perceptual account and then consider how personality traits may modulate these associations.

### 4.1 Classification of synesthesia: conceptual or perceptual

The results of our regression models suggest that the direction of associations with lucid-dreaming indices may differ across synesthesia subtypes. In particular, Type 3 (Spatial sequence) and Type 4 (Visualized sensation) showed broadly positive associations, whereas Type 1 (Language–color) and Type 2 (OLP) showed weaker associations, or even negative associations in some models. This pattern points to heterogeneity that cannot be fully explained by treating synesthesia as a single, unitary phenomenon. Accordingly, as a working hypothesis for interpreting our findings, we introduce a framework that organizes synesthesia types along a conceptual-perceptual dimension.

Here, “conceptual” refers to processing in which stimuli are identified by category or concepts (e.g., phoneme of a grapheme “a”) rather than by lower-level physical features (e.g., font or size of a grapheme “a”). In contrast, “perceptual” refers to processing closely tied to a stimulus's sensory characteristics and that can change dynamically in response to context.

Type 1 (Language–color) and Type 2 (OLP) are often induced by symbolic stimuli such as letters, numbers, and days of the week, and previous studies have reported that the induced experiences are largely unaffected by perceptual features such as font or size. Therefore, these types can be regarded as “conceptual synesthesia,” driven primarily by conceptual processing (Simner, 2012; Dixon et al., 2004; Rich et al., 2005; Witthoft and Winawer, 2006; Ramachandran and Hubbard, 2003; Chiou and Rich, 2014). For example, the tendency to select a common color for the Arabic numeral “1” and the Japanese numeral “؀” suggests that the determining factor may be the concept they represent rather than their visual form *per se* (Asano and Yokosawa, 2012).

By contrast, Type 4 (Visualized sensation) implies that perceptual inputs such as music, emotion, pain, and odors are accompanied by visual experiences (e.g., color, brightness, and shape). For example, studies of sound–color synesthesia have reported that the magnitude or brightness of synesthetic experiences varies continuously in response to continuously changing stimulus frequencies (Chiou et al., 2013; Ward et al., 2006). This suggests the involvement of processing tightly coupled to the stimulus's sensory properties. In this sense, Type 4 can be regarded as a prototypical form of more “perceptual” synesthesia.

Type 3 (Spatial sequence) may appear conceptual insofar as it is triggered by symbolic elements such as numbers, time, height, and temperature. However, because the experiential content involves a sequence being evoked as a continuous spatial layout, it has been suggested that the inducer may be represented not as a discrete concept but rather as a continuous magnitude, and may thus function as a stimulus with perceptual qualities (Price, 2014; Gould et al., 2014).

Nevertheless, the conceptual/perceptual positioning is not fixed. For instance, for children before learning letters, letters may be processed more as mere shapes, and thus more perceptually. Furthermore, even in sound–color synesthesia, some cases involve discrete pitch-class concepts acting as synesthetic inducers (e.g., C as red, D as yellow, E as green), rather than continuous acoustic properties. In such instances, the synesthetic association is likely to have a stronger conceptual component (Itoh et al., 2019). The conceptual/perceptual organization used in this study is therefore a pragmatic framework that presupposes such continuity. In the next subsection, we explore how this distinction between “more conceptual” and “more perceptual” synesthesia might relate to dream experiences—particularly controllable lucid dreams—from the perspectives of SMCs and counterfactual richness.

### 4.2 Impact of synesthesia types (conceptual/perceptual classification) on dream experiences

Based on the conceptual–perceptual distinction introduced in the previous section, we interpret the associations between synesthesia types and lucid-dream outcomes (LuCiD) (Tables 3, 4, Figure 2).

Type 4 (Visualized sensation), which is characterized by perceptual features, showed a positive association with the LuCiD total score and yielded consistently positive coefficients across a wide range of subscales, including Insight, Control, Thought, Realism, Memory, Dissociation, and Positive Emotion. One possible interpretation is that Type 4 involves dynamically generated visual concurrents (e.g., color, shape, brightness) in response to continuous sensory inputs such as sounds, odors, pains–that is, the repertoire of possible SMCs is continuously and dynamically generated –thereby providing excessive counterfactual-richness (O'Regan and Noë, 2001; Ikegami and Zlatev, 2008; Seth, 2014).

Following the perceptual-presence account outlined in the Introduction, the excessive counterfactual richness of Type 4 thereby enhances perceptual presence in dreams—that is, the sense that the dream world is “really there.” A heightened perceptual presence may, in turn, provide a more coherent and action-relevant world model during dreaming, facilitating metacognitive monitoring of the ongoing experience (e.g., noticing inconsistencies and reflecting on one's state) and intentional intervention. This interpretation offers a plausible bridge from SMC/counterfactual-richness to LuCiD facets such as Insight, Dissociation, and Control, and may also be consistent with more elaborate in-dream cognition and recall (e.g., Thought, Memory) and a more realistic phenomenology (Realism). We emphasize that these links remain theoretical and should be tested more directly in future work using measures that capture SMC/counterfactual structure and perceptual presence during dreaming.

Similarly, Type 3 (Spatial sequence) also showed positive associations with the LuCiD total score and, in particular, with Control, Dissociation, and Positive Emotion. Because Type 3 is experienced as a sequential spatial layout that emerges as a continuous configuration, it may more readily increase potential SMCs related to spatial aspects of dreaming, such as scene construction and self-localization.

In contrast, for the more conceptually dominant types–Type 1 (Language–color) and Type 2 (OLP)–associations with LuCiD outcomes were generally limited. For Type 1, negative coefficients were observed for Insight and Control, while a positive coefficient was observed for Negative Emotion. Moreover, the retention of interaction terms such as Type 1 × Extraversion, Type 2 × Extraversion, and Type 2 × Openness in the final models suggests that the influence of conceptual synesthesia may not be uniform but may instead be conditional on personality traits and/or cognitive style. Conceptual synesthesia relies on conceptual processing that can become highly automatized through learning (Stroop, 1935; MacLeod, 1991), and thus does not necessarily involve the sequential, continuous updating of SMCs assumed for perceptually grounded synesthesia. Within the present framework (SMC/counterfactual-richness), the absence of a consistent “enhancement” pattern for Type 1/2 across LuCiD facets can therefore be regarded as compatible with their concept-dominant and automatic-processing characteristics.

In summary, organizing synesthesia types along a perceptual (Visualized sensation, Spatial sequence) vs. conceptual (Language–color, OLP) dimension is useful for understanding their contributions to lucid-dream outcomes. At least for the perceptually grounded types (Type 3/4), the observed pattern was consistent with our hypothesis that increased counterfactual-richness may facilitate dream controllability and metacognitive facets of lucidity. This conceptual reclassification may also offer implications for the cognitive and phenomenological understanding of synesthesia. Future studies should incorporate indices that more directly capture SMC/counterfactual-richness and examine their influence on dreaming and other everyday experiences.

### 4.3 Role of personality traits in the synesthesia–lucid dream association

The regression models suggested that personality traits can contribute to LuCiD outcomes both as direct effects (main effects) and as moderating effects via interactions with synesthesia types (Tables 3, 4, Figure 2). Below, we focus on Extraversion, Openness, and Agreeableness and interpret their main effects and interaction patterns for the LuCiD subscales. Bivariate correlations (Table 2) are referenced only as supplementary information.

#### 4.3.1 Extraversion

Extraversion was retained with a positive coefficient for the LuCiD total score and also showed positive main effects for the subscales Insight, Control, Memory, and Positive Emotion (Tables 3, 4). Associations between Extraversion and lucid dreaming (and related dream indices) have been reported in prior work (Hess et al., 2017; Schredl et al., 2016), and the present results are broadly consistent with this literature.

Individuals higher in Extraversion tend to allocate more attention to positively valenced emotional information and show a positivity bias in memory (Haas and Canli, 2008; Dolcos et al., 2011). Autobiographical memory research further suggests that extraversion predicts recalling more positive personal events and may contribute to maintaining a positive mood after such recall (Denkova et al., 2012; Dolcos et al., 2011). From this perspective, higher Extraversion may be reflected in easier recall of dream experiences (Memory) and more positive evaluations of dream experiences (Positive Emotion). In addition, the active engagement that often accompanies Extraversion may contribute to self-reports of metacognitive awareness (Insight) and intentional dream manipulation (Control).

Importantly, however, the effect of Extraversion was moderated by synesthesia type. For the conceptually oriented Type 1 (Language–color), Type 1 × Extraversion was retained as a negative interaction for the LuCiD total score, and similar negative interactions were observed for Insight and Thought. For Type 2 (OLP), Type 2 × Extraversion was retained as a negative interaction for Control (Table 4). These patterns suggest that the positive contribution of Extraversion may be attenuated among individuals with conceptual synesthesia. Because the present study did not directly measure underlying processes, future research should test this possibility using more proximal indicators such as dream metacognition, attentional control, and imagery-manipulation strategies.

By contrast, for the perceptually oriented Type 4 (Visualized sensation), Type 4 × Extraversion was retained as a positive interaction for Thought and Negative Emotion (Table 4). Given that Extraversion showed no strong bivariate association with Negative Emotion (Table 2), this finding should not be interpreted as evidence that Extraversion generally increases negative dream affect. Rather, it may indicate that, specifically among individuals with richly perceptual synesthetic experiences, higher Extraversion is associated with increased dream-cognitive activity (Thought) and with a broader range of emotional arousal that can also influence reports of Negative Emotion.

#### 4.3.2 Openness

Openness is a trait that encompasses intellectual curiosity for novelty and complexity, imaginative engagement, and introspective attitudes, and prior studies have reported positive associations between Openness and lucid dreaming (Schredl et al., 2016; Hess et al., 2017). In the present study, Openness also showed small positive bivariate correlations with the LuCiD total score and with the Control and Thought subscales (Table 2). However, in the regression model including other personality traits and synesthesia types, the main effect of Openness did not remain significant for the LuCiD total score (Table 3), suggesting that its contribution may be shared with other predictors and may be relatively small as an independent effect.

Notably, Openness showed a positive main effect on the Thought subscale (Table 4). This may indicate that individuals high in Openness are more likely to report vivid, active cognitive activity in dreams. This interpretation is consistent with the idea that Openness-related imaginative engagement and associative richness are reflected in subjective evaluations of dream cognition (Thought). Relatedly, Openness has been reported to be positively associated with the subjective experience of autobiographical memory (e.g., vividness) and with the use of memory (Rasmussen and Berntsen, 2010), which is also compatible with an “access to internal experience” account.

Furthermore, because Type 2 (OLP) × Openness was retained as a negative coefficient for the LuCiD total score (and for Dissociation) (Tables 3, 4), Openness may be better conceptualized not as a uniform “enhancer” of lucid dreaming but as a moderating factor whose association with lucid-dream outcomes can be conditional on synesthesia type—particularly for conceptually oriented synesthesia.

#### 4.3.3 Agreeableness

In the present study, Agreeableness showed a negative main effect on Dissociation (i.e., the separation between self and dream content). Higher Agreeableness may be associated with stronger empathic attunement and involvement in dream events and emotions, resulting in lower reported Dissociation. In addition, for Negative Emotion, Type 4 (Visualized sensation) × Agreeableness was retained as a negative coefficient. From the perspective that Type 4 is counterfactually-rich, this interaction may suggest that even under conditions where perceptually rich experiences are more likely, Agreeableness (e.g., interpersonal harmony, empathy, and conflict-avoidance tendencies) may buffer emotional appraisal and reduce reports of negative dream affect.

#### 4.3.4 Summary

The explanatory power of the regression models was modest (adjusted *R*^2^≈0.10 for the total score and approximately 0.02–0.08 across subscales). Given that lucid-dream experiences are likely determined by multiple factors, synesthesia type and the Big Five may capture only a small portion of the variance. Moreover, because the present study is based on self-report data, recall and evaluative biases cannot be disentangled. Future research should employ prospective and repeated measurements (e.g., dream diaries) and explicitly incorporate mechanistically proximal variables (e.g., imagery vividness, absorption, dream-recall frequency, and metacognitive monitoring) to test reproducibility and to refine explanatory models.

### 4.4 Continuity hypothesis between synesthetic and dream experiences

The findings of the current study suggest the possibility of a continuous influence of synesthetic perceptual experiences extending into dream experiences, particularly lucid dreaming. This interpretation aligns with the Continuity Hypothesis (Schredl, 2003; Domhoff, 2017), which proposes that dream content is continuous with waking cognitive processing and everyday perceptual experiences. According to the Continuity Hypothesis, dreams reflect an extension of cognitive processes and sensory experiences that frequently occur in daily life.

Synesthetes engage in specific perceptual processes in response to particular stimuli (e.g., letters, numbers, sounds) during their waking lives. In particular, perceptual synesthetes (Visualized sensation, Spatial sequence) may inherently provide excessive sensorimotor contingencies (SMCs) to sensory stimuli. Such a perceptual processing style likely continues into dream experiences, potentially facilitating lucid dream experiences characterized by enhanced dream content control (due to excessive SMCs) and heightened perceptual presence. Consequently, these experiences may lead to increased insight (awareness of dreaming) and dissociation (the separation of self from dream content).

Conceptualizing synesthetic sensory-perceptual processing in daily life as potentially continuous with dream experiences may provide a more comprehensive and phenomenological account of the relationship between synesthesia and lucid dreaming. Future research should combine quantitative assessments of synesthesia (e.g., temporal-consistency measures indexing temporal stability) with phenomenological analyses of dream reports to clarify how everyday synesthetic experiences are reflected in dream content and dream awareness. Because the present study is based on self-report data, the mechanisms proposed here should be regarded as conceptual and interpreted with appropriate caution.

### 4.5 Methodological limitations and scope of interpretation

As anticipated from our a priori decision to use a broad self-report questionnaire (Rouw and Scholte, 2016), we observed a high endorsement rate of synesthetic experiences in this sample, which should not be interpreted as a prevalence estimate for the general population. Participants were recruited from a university course focused on “dreams and sleep,” a context that may have attracted students with a heightened interest in dreaming and related anomalous or introspective experiences. Moreover, because our synesthesia assessment relied on self-report rather than a behavioral consistency test, endorsement should be interpreted as indicating self-reported synesthesia-like experiences (or a broader synesthetic trait) rather than a definitive behavioral diagnosis of synesthesia. Accordingly, when compared with prevalence estimates for grapheme–color synesthesia reported in prior population-based studies (Simner and Hubbard, 2006; Ward, 2013), the elevated endorsement rate observed here likely reflects both sample-specific characteristics and the breadth of the self-report methodology (Eagleman et al., 2007; Rouw and Scholte, 2016; Ward and Simner, 2022). As noted in previous work, self-report approaches can capture a broader range of cross-modal experiences, including associations that may not meet strict temporal-consistency criteria (Rouw and Scholte, 2016; Ward and Simner, 2022).

Importantly, hierarchical clustering of item-level endorsements reproduced the targeted four-type structure in a manner broadly consistent with earlier self-report clustering studies (Ward and Simner, 2022), providing evidence that participants' responses were systematically structured rather than arbitrary. Nevertheless, this structural replication does not establish temporal consistency at the individual level; therefore, the present conclusions are restricted to associations between self-reported subtype endorsements and LuCiD outcomes. Notably, the relatively similar endorsement rates across the four targeted types (Figure 1a) are consistent with questionnaire-based clustering work: Ward and Simner (2022) reported broadly comparable cluster prevalences for Language–Color (0.684), Personification (0.440), Visualized sensations (0.576), and Sequence-space synesthesia (0.618).

Several additional limitations should be noted. First, both synesthesia and lucid dreaming were assessed retrospectively via self-report; individual differences in introspective tendency, response style, and dream recall could therefore inflate associations between subtype endorsement and LuCiD scores. Second, the cross-sectional design precludes causal inference: synesthesia-like experiences might facilitate lucid dreaming, lucid-dream propensity (or interest in dreaming) might increase synesthesia endorsement, or a third factor (e.g., imagery/absorption or metacognitive monitoring) may contribute to both.

In addition, the variance explained by the regression models was modest (adjusted *R*^2^ = 0.103 for the LuCiD total score; 0.024–0.076 across subscales), indicating small effect sizes and substantial unexplained variability. Finally, because models (including interaction terms) were selected via an AIC-based stepwise procedure across multiple outcomes, some retained effects may be sample-specific. Accordingly, the present findings should be treated as hypothesis-generating and should be tested in independent samples using preregistered analyses, together with prospective dream measures (e.g., diaries) and behavioral diagnostics of synesthesia.

Finally, the internal consistency of the LuCiD subscales should be considered when interpreting the present findings. Although the LuCiD total score showed adequate reliability (Cronbach's α = 0.79), several subscales–particularly Realism (α = 0.66), Memory (α = 0.62), and Dissociation (α = 0.69)–exhibited relatively lower internal consistency. These subscales—Realism, Memory and Dissociation—may be particularly sensitive to state-dependent fluctuations and individual differences in metacognitive access to dream experiences, which could reduce item homogeneity. While these values fall within ranges commonly considered acceptable for psychological research, reduced reliability may increase measurement noise and attenuate effect estimates, especially in regression analyses. Accordingly, the results for these subscales should be interpreted with appropriate caution. Future research should re-examine item wording, assess test–retest reliability, and consider confirmatory factor analytic approaches to further refine the measurement properties of the LuCiD in relation to synesthesia research.

Finally, recent phenomenological work suggests that synesthetic experiences may involve dynamically generated sensory–affective qualities that are not fully captured by temporal consistency measures alone (Matsuda and Daikoku, 2025). Future research should therefore combine behavioral diagnostics (e.g., consistency testing) with self-report and phenomenological approaches to clarify which facets of synesthetic experience are most relevant to lucid-dreaming outcomes.

## 5 Conclusion

This study demonstrated a close association between synesthesia and lucid dreaming experiences through the concept of counterfactual-richness. Specifically, perceptual synesthesia types (Spatial sequence and Visualized sensation) were shown to significantly enhance various aspects of lucid dreaming, including dream control, insight, and dissociation. In contrast, conceptual synesthesia types (Language-color and OLP) exhibited inhibitory effects, providing novel insights into synesthesia classification and underlying mechanisms.

In this study, a self-report method was used to evaluate synesthesia comprehensively. However, future research should employ standard diagnostic methods (e.g., temporal consistency tests) to identify synesthetes rigorously, and subsequently conduct in-depth qualitative analyses of the relationship between synesthesia and lucid dreaming using smaller samples. In particular, future investigations should focus on Spatial sequence and Visualized sensation synesthesia types, which exhibit pronounced associations with lucid dreaming, and examine how these types qualitatively influence dream content. Such studies would deepen our understanding of the synesthesia-dream relationship and provide a more concrete empirical evaluation of the concept of counterfactual-richness.

## Data Availability

The original contributions presented in the study are included in the article/Supplementary material, further inquiries can be directed to the corresponding author.
