# Supplementary material for: Exploring the link between synesthesia and lucid dreaming through perceptual presence
Source: Front Psychol. 2026 Feb 26;17:1733841. doi: 10.3389/fpsyg.2026.1733841 (PMC12979406; doi:10.3389/fpsyg.2026.1733841)
Supplement: Supplementary file 1 [file Data_Sheet_1.pdf]

Supplementary Material

1 SUPPLEMENTARY TABLES AND FIGURES

1.1 Tables

Table S1. Self-report questionnaire on synesthetic experiences (adapted from the self-report survey framework of Ward and Simner (2022)). For the main analyses, a participant was coded as endorsing a given type if they answered "Yes" and selected at least one inducer–concurrent option within that type. Participants could endorse multiple types. Items labeled "Assessed; not included in main analyses" were included to retain the broader coverage of synesthetic experiences in the Ward and Simner (2022) questionnaire framework (including less prevalent forms), but were excluded from the main inferential analyses due to low endorsement rates and limited statistical power.

1. Do you experience colors associated with letters, numbers, or words? Yes / No [Type 1: Language–color]
   If yes, select all that apply:
   Numbers · Letters · Days of the week · Months · People's names · English words · Japanese words · Words in other languages · Phonetic symbols

2. Do you experience colors associated with sensory stimuli (e.g., sounds, pain, taste)? Yes / No [Type 4: Visualized sensation]
   If yes, select all that apply:
   Music · Voice · Noise · Emotions · Pain · Touch · Smell · Taste · Posture

3. Do you experience shapes associated with sensory stimuli (e.g., sounds, pain, taste)? Yes / No [Type 4: Visualized sensation]
   If yes, select all that apply:
   Music · Voice · Noise · Emotions · Pain · Touch · Smell · Taste

4. Do you experience tastes associated with letters, numbers, or words? Yes / No [Assessed; not included in main analyses]
   If yes, select all that apply:
   Numbers · Letters · Days of the week · Months · People's names · English words · Japanese words · Words in other languages

5. Do you perceive sequences (e.g., time, numbers) as spatially arranged? Yes / No [Type 3: Spatial sequence]
   If yes, select all that apply:
   Numbers · Letters · Days of the week · Months · Years · Temperature · Height · Weight

6. Do you experience personality or gender associated with letters, numbers, or words? Yes / No [Type 2: OLP / Personification]
   If you experience personality, select all that apply:
   Numbers · Letters · Days of the week · Months
   If you experience gender, select all that apply:
   Numbers · Letters · Days of the week · Months

7. Do you feel another person's pain or touch as if it were your own? Yes / No [Assessed; not included in main analyses]
   If yes, select all that apply:
   Pain · Touch

8. Do you experience heard words as visually written (like subtitles)? Yes / No [Assessed; not included in main analyses]
   If yes, do these words have associated colors? Yes / No

9. Do you experience sounds associated with moving objects (even if they do not produce actual sounds)? Yes / No [Assessed; not included in main analyses]

10. Please describe any other types of synesthetic experiences you have that are not listed above. [Open-ended]

Table S2. Results of multiple regression analyses for LuCiD subscales (Part 1 of 2: Insight–Realism). Predictors retained in the final models were selected using an AIC-based stepwise procedure; therefore, coefficients are reported for all retained predictors, including non-significant terms. All variables were standardized.

Dependent Variable	Predictor	Estimate	Std. Error	t-value	p-value
**Insight (d1)**	Type 1 (Language–color; s1)	-0.0918	0.0403	-2.278	0.023*
	Type 3 (Spatial sequence; s3)	0.0770	0.0409	1.885	0.060.
	Type 4 (Visualized sensation; s4)	0.1517	0.0414	3.662	< 0.001***
	Extraversion (p1)	0.0821	0.0398	2.060	0.040*
	Conscientiousness (p3)	0.0625	0.0395	1.581	0.114
	Openness (p5)	0.0434	0.0393	1.104	0.270
	Type 1 × Extraversion (s1:p1)	-0.1074	0.0394	-2.729	0.007**
	Type 4 × Openness (s4:p5)	-0.0715	0.0399	-1.794	0.073.
Adjusted R^2	0.061				
**Control (d2)**	Type 1 (s1)	-0.0886	0.0404	-2.194	0.029*
	Type 2 (OLP; s2)	-0.0187	0.0416	-0.450	0.653
	Type 3 (s3)	0.1026	0.0414	2.481	0.013*
	Type 4 (s4)	0.1975	0.0417	4.739	< 0.001***
	Extraversion (p1)	0.0973	0.0393	2.474	0.014*
	Openness (p5)	0.0613	0.0391	1.569	0.117
	Type 2 × Extraversion (s2:p1)	-0.0807	0.0377	-2.139	0.033*
	Type 4 × Openness (s4:p5)	-0.0688	0.0394	-1.745	0.081.
Adjusted R^2	0.076				
**Thought (d3)**	Type 1 (s1)	-0.0150	0.0400	-0.374	0.709
	Type 3 (s3)	0.0507	0.0406	1.249	0.212
	Type 4 (s4)	0.1852	0.0413	4.489	< 0.001***
	Extraversion (p1)	0.0699	0.0394	1.775	0.076.
	Openness (p5)	0.0909	0.0390	2.331	0.020*
	Type 1 × Extraversion (s1:p1)	-0.1066	0.0405	-2.633	0.009**
	Type 3 × Extraversion (s3:p1)	-0.0713	0.0431	-1.653	0.099.
	Type 4 × Extraversion (s4:p1)	0.0930	0.0419	2.220	0.027*
Adjusted R^2	0.073				
**Realism (d4)**	Type 1 (s1)	0.0763	0.0409	1.865	0.063.
	Type 3 (s3)	0.0211	0.0415	0.508	0.612
	Type 4 (s4)	0.1047	0.0419	2.501	0.013*
	Neuroticism (p4)	0.0590	0.0400	1.477	0.140
	Type 3 × Neuroticism (s3:p4)	-0.0804	0.0413	-1.947	0.052.
Adjusted R^2	0.024				

Note: . $p < 0.1$, * $p < 0.05$, ** $p < 0.01$, *** $p < 0.001$.

Table S3. Results of multiple regression analyses for LuCiD subscales (Part 2 of 2: Memory–Positive Emotion). Predictors retained in the final models were selected using an AIC-based stepwise procedure; therefore, coefficients are reported for all retained predictors, including non-significant terms. All variables were standardized.

Dependent Variable	Predictor	Estimate	Std. Error	t-value	p-value
**Memory (d5)**	Type 1 (s1)	-0.0239	0.0400	-0.597	0.551
	Type 3 (s3)	0.0753	0.0405	1.857	0.064.
	Type 4 (s4)	0.1800	0.0412	4.369	< 0.001***
	Extraversion (p1)	0.1234	0.0393	3.136	0.002**
	Openness (p5)	0.0727	0.0390	1.861	0.063.
	Type 1 × Extraversion (s1:p1)	-0.0715	0.0391	-1.829	0.068.
Adjusted R^2	0.071				
**Dissociation (d6)**	Type 2 (s2)	0.0079	0.0414	0.190	0.849
	Type 3 (s3)	0.0929	0.0415	2.238	0.026*
	Type 4 (s4)	0.1784	0.0414	4.311	< 0.001***
	Extraversion (p1)	0.0634	0.0404	1.569	0.117
	Agreeableness (p2)	-0.0889	0.0407	-2.185	0.029*
	Neuroticism (p4)	-0.0490	0.0415	-1.180	0.239
	Openness (p5)	-0.0205	0.0397	-0.518	0.605
	Type 4 × Neuroticism (s4:p4)	-0.0689	0.0397	-1.736	0.083.
	Type 2 × Openness (s2:p5)	-0.1187	0.0403	-2.947	0.003**
Adjusted R^2	0.067				
**Negative Emotion (d7)**	Type 1 (s1)	0.0966	0.0414	2.334	0.020*
	Type 2 (s2)	-0.0232	0.0418	-0.554	0.580
	Type 4 (s4)	0.0591	0.0423	1.397	0.163
	Extraversion (p1)	-0.0270	0.0409	-0.661	0.509
	Agreeableness (p2)	-0.0209	0.0406	-0.514	0.607
	Conscientiousness (p3)	-0.0680	0.0405	-1.680	0.093.
	Type 2 × Extraversion (s2:p1)	0.0664	0.0397	1.671	0.095.
	Type 4 × Extraversion (s4:p1)	0.0887	0.0408	2.175	0.030*
	Type 4 × Agreeableness (s4:p2)	-0.0842	0.0408	-2.063	0.040*
	Type 1 × Conscientiousness (s1:p3)	0.0576	0.0408	1.412	0.158
Adjusted R^2	0.030				
**Positive Emotion (d8)**	Type 1 (s1)	-0.0151	0.0406	-0.372	0.710
	Type 3 (s3)	0.1144	0.0411	2.786	0.006**
	Type 4 (s4)	0.1456	0.0416	3.499	< 0.001***
	Extraversion (p1)	0.0926	0.0403	2.298	0.022*
	Agreeableness (p2)	-0.0014	0.0401	-0.036	0.971
	Conscientiousness (p3)	0.0339	0.0399	0.850	0.396
	Openness (p5)	-0.0301	0.0395	-0.762	0.446
	Type 1 × Extraversion (s1:p1)	-0.0638	0.0397	-1.608	0.108
	Type 4 × Agreeableness (s4:p2)	0.0757	0.0405	1.871	0.062.
	Type 4 × Conscientiousness (s4:p3)	-0.0674	0.0400	-1.686	0.092.
	Type 1 × Openness (s1:p5)	-0.0691	0.0394	-1.757	0.079.
Adjusted R^2	0.055				

Note: . $p < 0.1$, * $p < 0.05$, ** $p < 0.01$, *** $p < 0.001$.

1.2 Figures

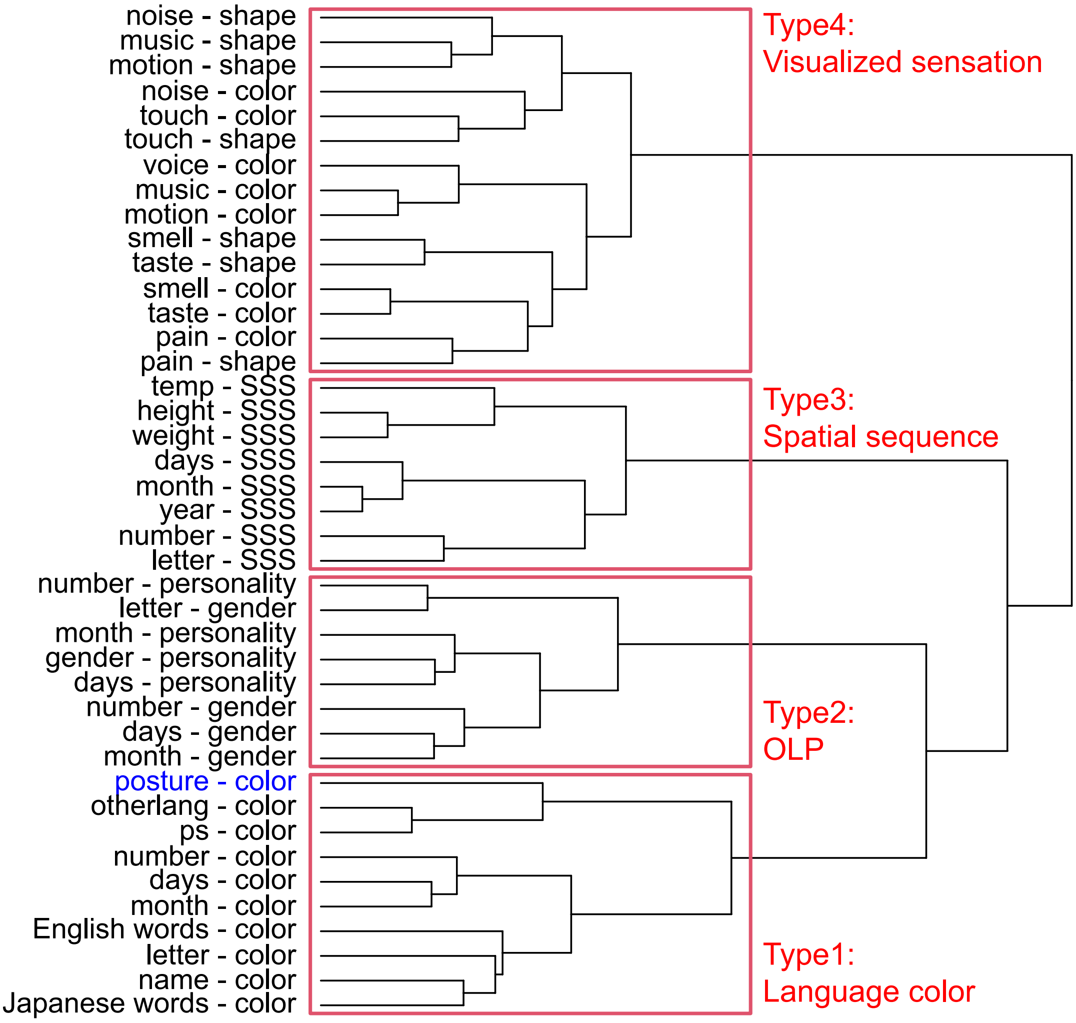

Figure S1. Dendrogram illustrating hierarchical cluster analysis (Ward's method; Ward.D2) of synesthetic items. The number of clusters was set to four following previous research. Each red box indicates one of the four clusters: Type 1 (Language-color), Type 2 (Ordinal Linguistic Personification; OLP), Type 3 (Spatial sequence), and Type 4 (Visualized sensation). The item labeled "Posture–color" (shown in blue) was classified within Type 1 despite its original properties, likely due to ambiguous interpretation of the item's meaning or weak associations with other items.
